# The therapeutic targets and signaling mechanisms of ondansetron in the treatment of critical illness in the ICU

**DOI:** 10.3389/fphar.2024.1443169

**Published:** 2024-08-21

**Authors:** Lili Tao, Zhenhui Zhang, Chuang Li, Minxuan Huang, Ping Chang

**Affiliations:** ^1^ Department of Critical Care Medicine, Zhujiang Hospital, Southern Medical University, Guangzhou, Guangdong, China; ^2^ Department of Critical Care Medicine, The Second Affiliated Hospital, Guangzhou Medical University, Guangzhou, Guangdong, China; ^3^ Department of Emergency Department, The Second Affiliated Hospital, Guangzhou Medical University, Guangzhou, Guangdong, China

**Keywords:** ondansetron, AKI, sepsis, ARDS, network pharmacology, NETs

## Abstract

**Background:**

There is accumulating evidence regarding the benefits of the 5-HT_3_ receptor antagonist ondansetron for the treatment of critical illness due to its potential anti-inflammatory effect. This study attempted to determine the potential targets and molecular mechanisms of ondansetron’s action against critical illnesses.

**Methods:**

A bioinformatics analysis of network pharmacology was conducted to demonstrate screening targets and the signaling pathways of ondansetron action against the most common critical illnesses such as acute kidney injury (AKI), sepsis, and acute respiratory distress syndrome (ARDS). Experiments of LPS-stimulated rat neutrophils with ondansetron treatment were conducted to further validate the relevant hypothesis.

**Results:**

A total of 198, 111, and 26 primary causal targets were identified from the data for the action of ondansetron against AKI, sepsis, and ARDS respectively. We found that the pathway of neutrophil extracellular traps (NETs) formation is statistically significantly involved in the action of ondansetron against these three critical illnesses. In the pathway of NETs formation, the common drug-disease intersection targets in these three critical illnesses were toll-like receptor 8 (*TLR8*), mitogen-activated protein kinase-14 (*MAPK14*), nuclear factor kappa-B1 (*NFKB1*), neutrophil elastase (*NE*), and myeloperoxidase (*MPO*). Considering these bioinformatics findings, we concluded that ondansetron anti-critical illness effects are mechanistically and pharmacologically implicated with suppression of neutrophils-associated inflammatory processes. It was also showed that after treatment of LPS-stimulated rat neutrophils with ondansetron, the key proteins NE, MPO, and Peptide Arginine Deaminase 4 (PAD4) in the NETs formation were significantly reduced, and the inflammatory factors IL-6, IL-1β, TNF-α, and chemokine receptor (CXCR4) were also significantly decreased.

**Conclusion:**

The excessive formation of NETs may have important research value in the development and progression of critical illness. Ondansetron may reduce excessive inflammatory injury in critical diseases by reducing the formation of NETs via influencing the five targets: *TLR8, NFKB1, MAPK14, NE, and MPO*. Ondansetron and these primary predictive biotargets may potentially be used to treat critical illness in future clinical practice.

## Introduction

For critically ill patients, excessive inflammatory response caused by massive neutrophil infiltration is one of the important clinical characteristics (Grégoire et al., 2018). Unbalanced immune response may lead to dysregulation of neutrophil extracellular traps (NETs) release, which will exacerbate inflammation and host tissue damage beyond antibacterial function of NETs (Hidalgo et al., 2022). Studies have shown that exaggerated release of neutrophil extracellular traps (NETs) along with decreased NETs clearance may contribute to sustained inflammation in acute respiratory distress syndrome (ARDS) (Grégoire et al., 2018).

In recent years, the 5-hydroxytryptamine 3 (5-HT_3_) receptor antagonist can be used as an important agent for potential intervention in the regulation of inflammatory disorders. For example, they were reported to exhibit an anti-inflammatory effect in chronic inflammatory diseases in experimental colitis models with decreased neutrophil infiltration, lipid peroxidation, and colonic inflammatory cytokines (Mousavizadeh et al., 2009; Rahimian et al., 2013; Rahimian et al., 2016). Moreover, they could effectively control the production of proinflammatory cytokine expression by macrophages in the pathogenesis of severe sepsis/septic shock in a mouse model study and protected mice against sepsis-induced death (Gong et al., 2019).

Ondansetron, the earliest 5-HT_3_ receptor antagonist, is a widely used antiemetic drug and a highly safe medication even for pregnant women (Sakran et al., 2021; Suarez et al., 2021). Our team previously found that ondansetron use was associated with a reduced risk of death in critical patients with AKI in the MIMIC-IV database (Tao et al., 2021). Thereafter, other scholars have further confirmed that daily low-to-moderate dose (0–16 mg) of early ondansetron application (from 24 h before ICU admission to 48 h after ICU admission) is protective against in-hospital mortality in ICU patients (Fang et al., 2022). Furthermore, we also found that the neutrophil–lymphocyte ratio may play substantial roles in the relationship between ondansetron treatment and the decreased mortality of ICU patients on mechanical ventilation (Zhou et al., 2022). Nevertheless, the mechanism by which ondansetron has therapeutic effects on critical illnesses is not yet clear.

This study was designed to determine the therapeutic targets and signaling mechanisms of ondansetron activity against the three most common ICU syndromes including AKI, sepsis, and ARDS through the method of network pharmacology and validated the related hypothesis by conducting cellular experiments.

## Methods

### Screening of anti-critical illness targets of ondansetron

Using the Traditional Chinese Medicine Systems Pharmacology (TCMSP) (Ru et al., 2014), Drugbank (Wishart et al., 2018), SuperPred (Gallo et al., 2022; Nickel et al., 2014), Swiss Target Prediction databases (Gfeller et al., 2014) and Pharmmapper (Wang et al., 2017), well-reported pharmacological targets of ondansetron were obtained. Likewise, the DisGeNET (Piñero et al., 2015), Drugbank, TCMSP, and Genecard databases (Fishilevich et al., 2017) were used to screen pathological targets of critical illness including AKI, sepsis, and ARDS. All primary targets of ondansetron and critical illness were evaluated using Venn diagrams to identify potential targets for ondansetron against critical illness (Zhou et al., 2019).

### Construction of interrelated network

Using a STRING database, the mapped target of ondansetron action against critical illnesses were re-assayed to harvest a target-to-target function-related protein network and protein–protein interaction (PPI) network.

### Assays of functional processes and molecular pathways

The R packages of “ClusterProfiler” were used for enrichment analysis of the Kyoto Encyclopedia of Genes and Genomes (KEGG) pathway using previously identified targets. This data was used to generate a corresponding bubble chart and histogram (Yu et al., 2012). KEGG analyses were performed using cluster Profiler (version 3.14.3) from the R packages. KEGG data were acquired from org. Hs. eg. Db using a cut-off value adjusted to *P* < 0.05.

### LPS-stimulated rat neutrophils treated with ondansetron

This study was approved by the Institutional Animal Care and Use Committee of the second affiliated hospital of Guangzhou Medical university. All animal experiments were performed according to the guidelines of the Animal Welfare Act and the Guide for Care.

### Isolation of neutrophils

Neutrophils were isolated from the blood from cardiac puncture of SD (Sprague Dawley) rats (GUANGDONG MEDICAL LABORATORY ANIMAL CENTER) by density gradient (Percoll) centrifugation techniques with a Neutrophil isolation kit (Bei jing Solarbio Science and Technology Co., Ltd.). Neutrophils were detected as double positive events for CD11b (PerCP-Cy5.5) and Ly6G (PE), and neutrophils purity was more than 80% via flow cytometry analysis. Neutrophils viability measured by Trypan Blue staining was more than 90%.

### Experimental design

Rat neutrophils were divided into three groups: blank control group, lipopolysaccharide (LPS) (1ug/mL) group and LPS group with ondansetron (1 μmol/L) treatment group(LPS + O). The three groups of rat neutrophils treated with LPS or ondansetron were incubated at 37°C for 4 h respectively. The following indicators for three groups were detected separately: 1) Inflammatory factors including interleukin-6 (IL-6) and interleukin-1β (IL-1β), tumor necrosis factor-α (TNF-α), and chemokine receptor 4 (CXCR4) (Malvoisin et al., 2011; Reutersberg et al., 2023; Kim et al., 2023; Choi et al., 2016); and 2) The key proteins in NETs formation including neutrophil elastase (NE), myeloperoxidase (MPO), and peptidylarginine deaminase 4 (PAD4).

### Determination of IL-6, IL-1β, TNF-α, CXCR4 and the key proteins levels in NETs formation

IL-6, IL-1β, TNF-α, CXCR4, MPO, NE, and PAD4 levels were determined using ELISA Kits (QUANZHOU RUIXIN BIOTECHNOLOGY CO., LTD.) according to the manufacturer’s instructions. The cells were incubated with LPS or Ondansetron (MEC, MedChemExpress), and the supernatants were assayed for IL-6, IL-1β, TNF-α, CXCR4, MPO, NE and PAD4 levels.

### Statistical analyses

Data are presented as means ± standard deviation (SD) for three independent experiments. Statistical analyses were performed using GraphPad 8 (GraphPad Software, San Diego, CA, United States). Means of two groups were compared using Student’s t-test. *P* < 0.05 was considered statistically significant. *P < 0.05, **P < 0.01, ***P < 0.001, and****P < 0.0001.

## Results

**FIGURE 1:**
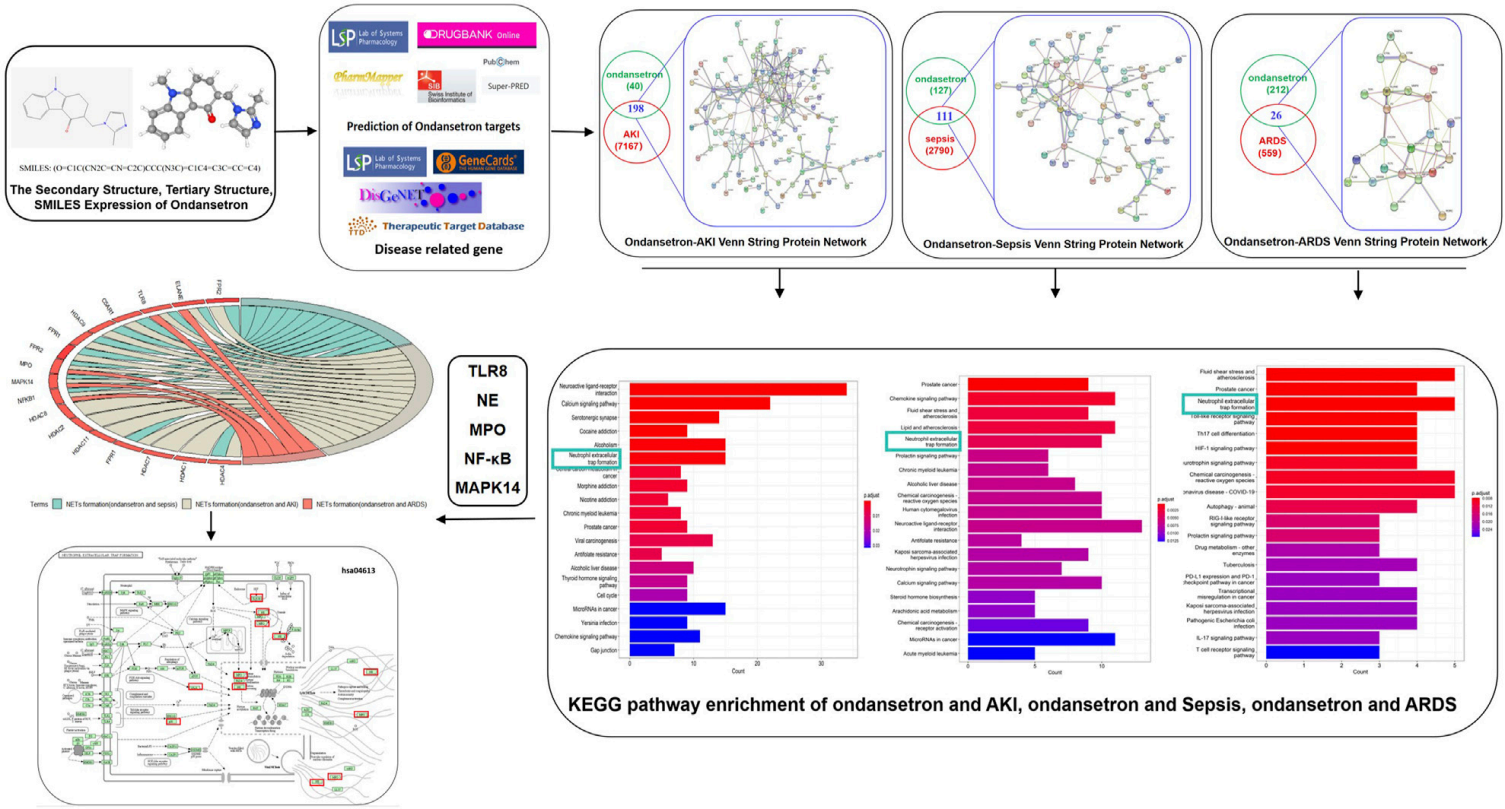
Investigative flowcharts for biotargets and signaling pathways of ondansetron’s action against critical illness through a systematic network pharmacology approach. All the reported genes/targets of ondansetron and critical illness were screened and identified using databases available online prior to harvesting the anti-critical illness targets of ondansetron. After the construction of a PPI network and identification of the targets of ondansetron against critical illness, the KEGG signaling pathways were determined. A network of ondansetron-target-KEGG-critical illness was visualized following bioinformatics analysis.

A bioinformatics diagram through systematic pharmacology approach that was used to help identify the ondansetron action against critical illness is shown in Figure 1.

### Detailed data of targets and PPI network

**FIGURE 2:**
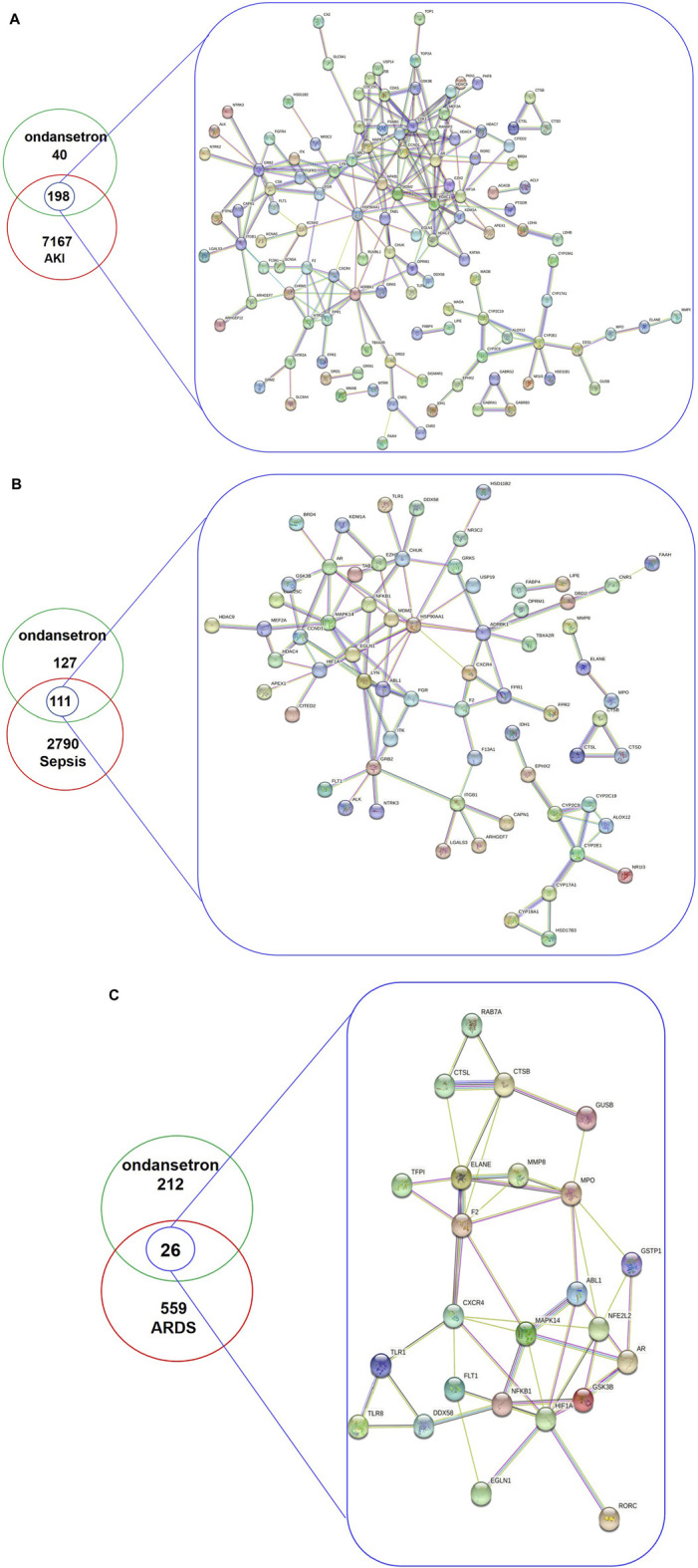
The total targets of ondansetron, AKI **(A)**, sepsis **(B)**, ARDS **(C)**, and intersection targets were identified using the Venn diagram and PPI network. In the analyses, the inclusion criteria for AKI, sepsis, and ARDS targets using DisGeNET and GeneCards databases were identified with gene-disease association scores of >0.01 and >0 1, respectively. A PPI network of the 198 **(A)**, 111 **(B)**, and 26 **(C)** targets was plotted following a STRING database with the interaction score set to confidence at 0.400.

The inclusion criteria for critical illness screening through DisGeNET database was a gene-disease association score >0.01 and for screening through GeneCard critical illness database was a gene score >0.1. Using these criteria, after removing duplicates, 7,367, 2,901, and 585 genes associated with AKI, sepsis, and ARDS, respectively, were identified. Ondansetron drug targets were screened and identified through the TCMSP, SuperPred, Pharmmapper, and Swiss Target Prediction databases; 238 ondansetron targets were identified (after removing duplicates) using the Uniprot database. A Venn diagram of the drug-disease target sets was then used to obtain 198, 111, and 26 intersection targets, respectively (Supplementary Table S1). Using a minimum required interaction score set to 0.400, a STRING database was used to collect function-related PPI data from the 198, 111, and 26 targets. The pharmacological targets of ondansetron action against critical illness and function-related PPI network were then constructed (Figure 2).

### Targets in the KEGG pathway enrichment

**FIGURE 3:**
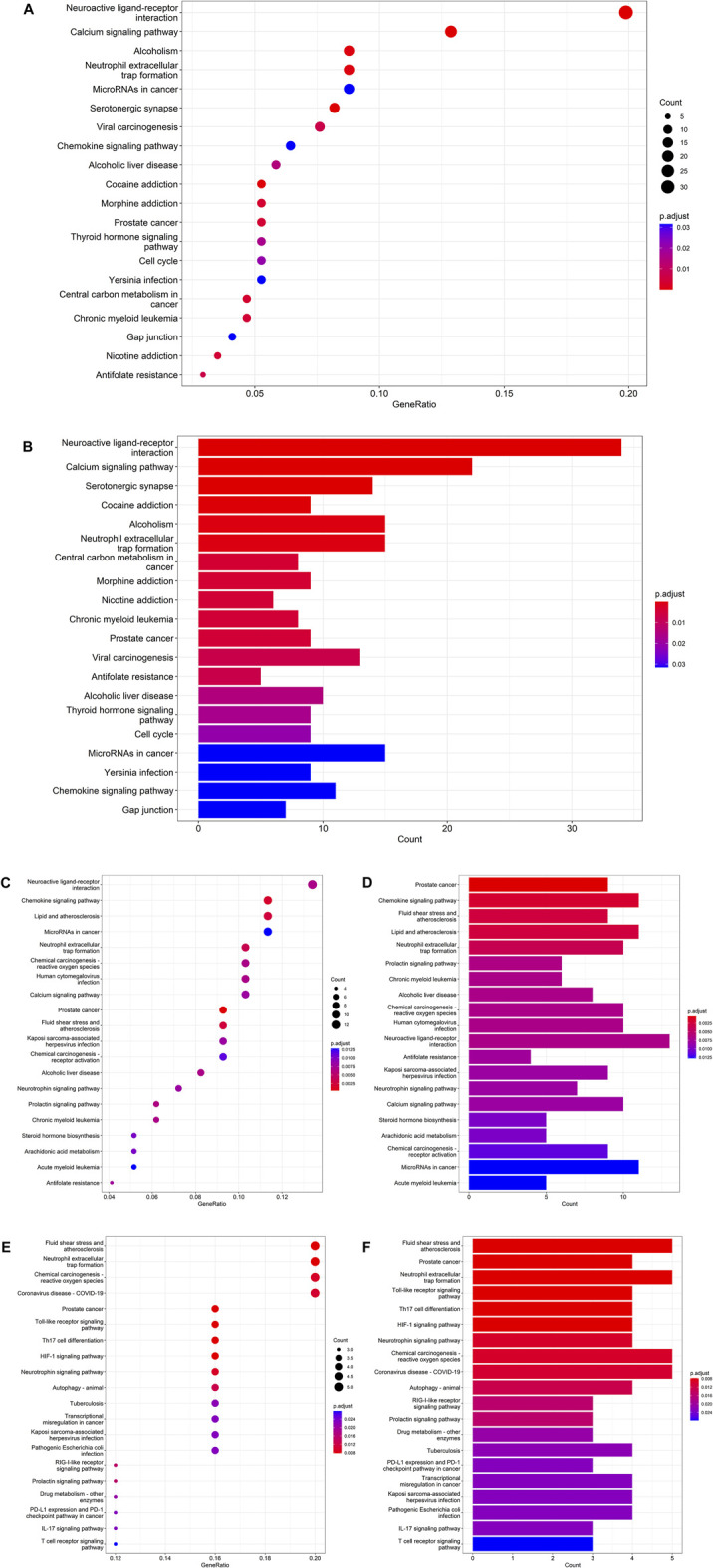
Detailed molecular pathways from bioinformatics findings exhibited the top 20 KEGG pathways of ondansetron action against AKI **(A,B)**, sepsis **(C,D)**, and ARDS **(E,F)**. Using the R-language packages, the drug-disease targets were reanalyzed to reveal the KEGG molecular pathways. **(A,C,E)** The bubble diagram shows the top 20 KEGG enrichment pathways; the *x*-axis represents the gene ratio and the intensities of different colors represent the adjusted *P*-value. **(B,D,F)** The histograms highlight the top 20 KEGG enrichment pathways. The *x*-axis represents the enriched gene count and the intensities of different colors represent the adjusted *P*-value.

Using R language-related packages, the targets were analyzed by KEGG pathway analysis. The results of these analyses were visualized using an enriched KEGG pathways bubble diagram and histogram (Figure 3). (Ondansetron and AKI: Figures 3A, B; Ondansetron and sepsis: Figures 3C, D; Ondansetron and ARDS: Figures 3E, F)

There were 198 intersection targets between the pharmacological targets of ondansetron and pathological targets of AKI. After enrichment analysis of the KEGG pathway, the results suggested that ondansetron may affect AKI by affecting NETs formation. This pathway ranks sixth in the top 20 pathways (P. adjust = 0.0004) (Supplementary Table S2; Supplementary Figure S1). The following 15 drug-disease intersection targets were enriched in the NETs formation pathways: *HDAC8, NFΚB1, HDAC4, TLR8, C5AR1, HDAC9, HDAC2, HDAC11, FPR1, FPR2, HDAC7, MPO, MAPK14, HDAC1, and ELANE (NE)*.

There were 111 intersection targets between pharmacological targets of ondansetron and pathological targets of sepsis. After enrichment analysis of the KEGG pathway, the results suggested that ondansetron may affect sepsis by affecting NETs formation. This pathway ranks fifth in the top 20 pathways (P. adjust = 0.0036) (Supplementary Table S3; Supplementary Figure S1). The following 10 drug-disease intersection targets were enriched in the NETs formation pathways: *NFΚB1, HDAC4, TLR8, C5AR1, HDAC9, FPR1, FPR2, MPO, MAPK14, and ELANE (NE)*.

There were 26 intersection targets between the pharmacological targets of ondansetron and pathological targets of ARDS. After enrichment analysis of the KEGG pathway, the results suggested that ondansetron may affect ARDS by affecting NETs formation. This pathway ranks third in the top 20 pathways (P. adjust = 0.0077) (Supplementary Table S4; Supplementary Figure S1). The following five drug-disease intersection targets were enriched in the NETs formation pathways: *NFKB1, TLR8, MPO, MAPK14, and ELANE (NE)*.

**FIGURE 4:**
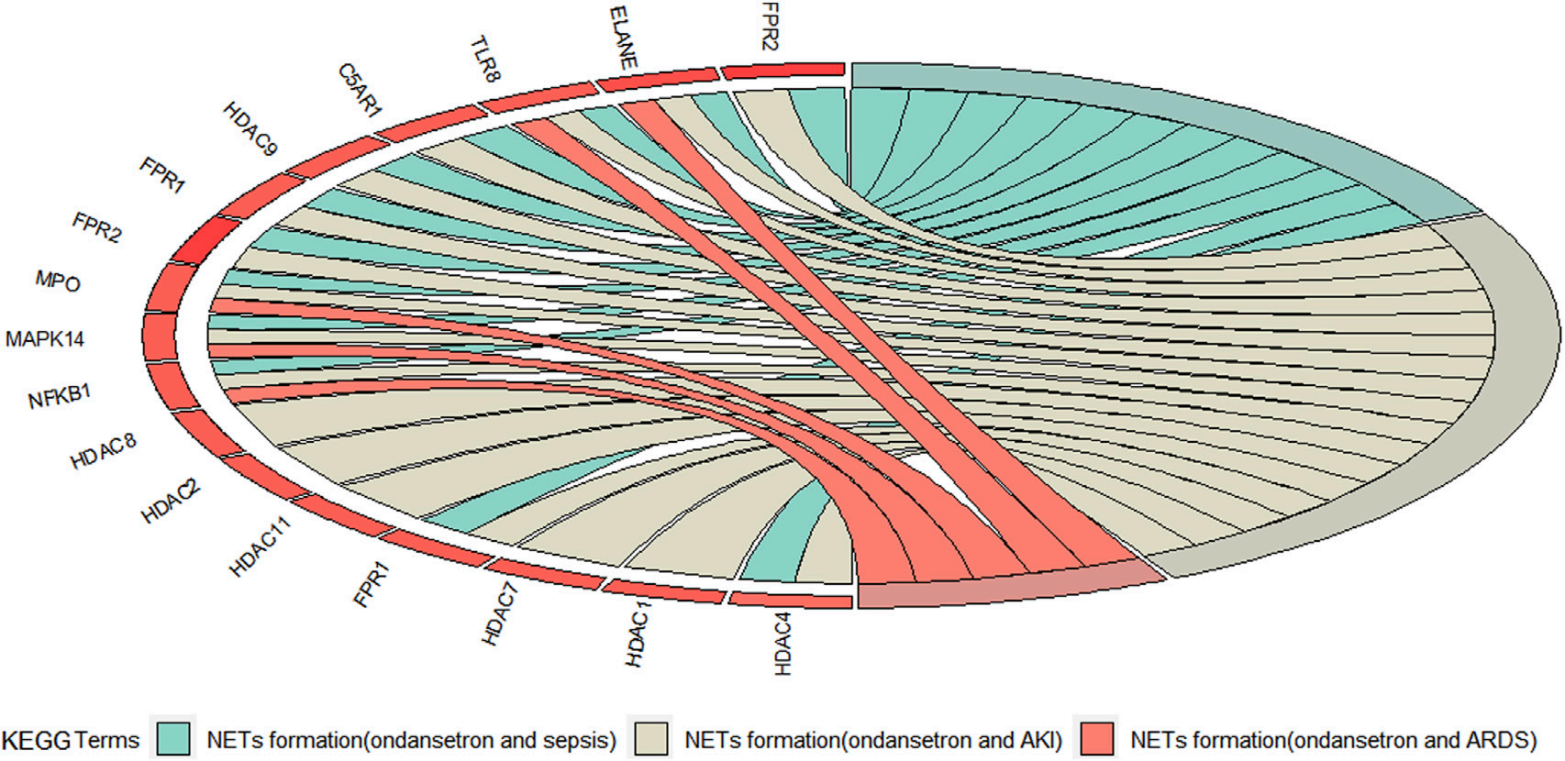
The red Circro circles show the genes enriched in the neutrophil extracellular traps (NETs) formation pathways of ondansetron’s action against AKI, sepsis, and ARDS.

After enrichment analysis of the KEGG pathway, the intersection targets in the action of ondansetron against these three critical conditions are all statistically significantly involved in the NETs formation pathway. Moreover, the NETs formation pathway is only common one in the top 20 pathways of ondansetron against AKI, sepsis, and ARDS (Supplementary Figures S1, S2). In the NETs pathways, the following five genes are the common intersection targets in the three ICU conditions: *TLR8, NFΚB1, MAPK14, ELANE (NE), and MPO* (Figure 4).

### Establishing scientific hypotheses

**FIGURE 5:**
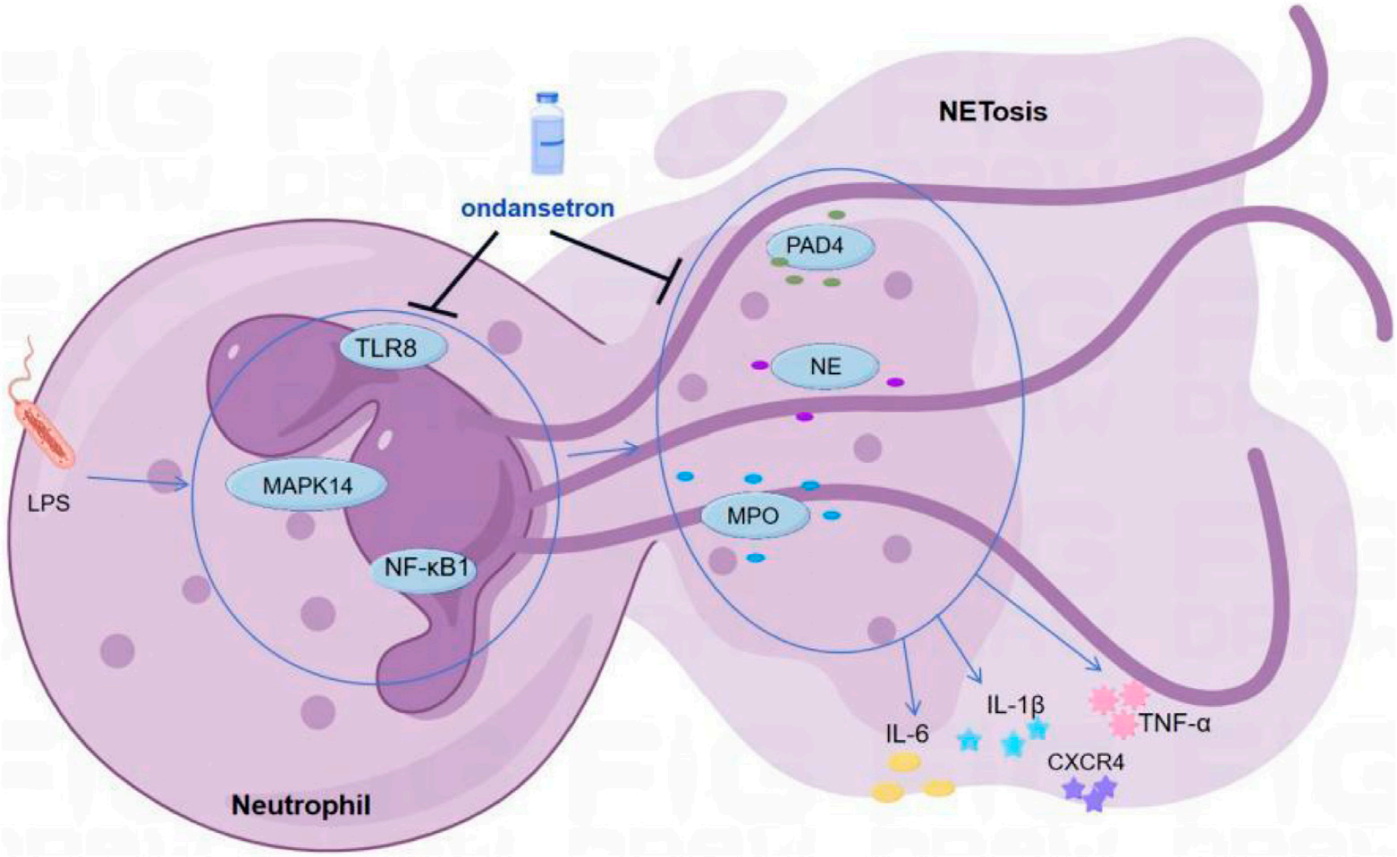
Scientific hypotheses. Ondansetron may reduce inflammatory injury in patients with critical illness by inhibiting the formation of NETs.

Considering the bioinformatic findings, we concluded that ondansetron may reduce inflammatory injury in patients with critical illness by inhibiting the formation of NETs. Ondansetron may affect *TLR8, NFKB1, MAPK14, NE, and MPO* to inhibit the formation of NETs (Figure 5).

### LPS-stimulated rat neutrophils treated with ondansetron

**FIGURE 6:**
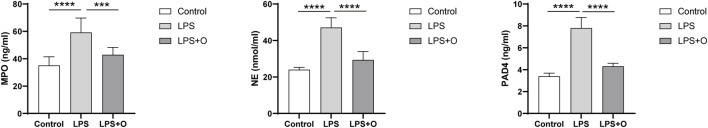
Ondansetron can decrease the core substances of NETs produced by neutrophils stimulated by LPS. Levels of the core substances NE, MPO, and PAD4 after being incubated at 37°C for 4 h in the cell supernatant of the control group, LPS group, and LPS + O group. Control, Neutrophils; LPS, Neutrophils stimulated by LPS; LPS + O, Neutrophils stimulated by LPS were treated with ondansetron. NE, neutrophil elastase; MPO, myeloperoxidase; PAD4, peptide arginine deaminase 4. Results are presented as mean ± SD. Student’s t-test was used to compare means of two groups. (***P < 0.001, ****P < 0.0001).

Compared with the control group, LPS stimulation of neutrophils resulted in a significant increase in the key proteins NE, MPO, and PAD4 produced by neutrophil NETs formation. Ondansetron treatment of LPS-stimulated neutrophils resulted in significantly reduced NE, MPO, and PAD4 levels (Figure 6).

**FIGURE 7:**
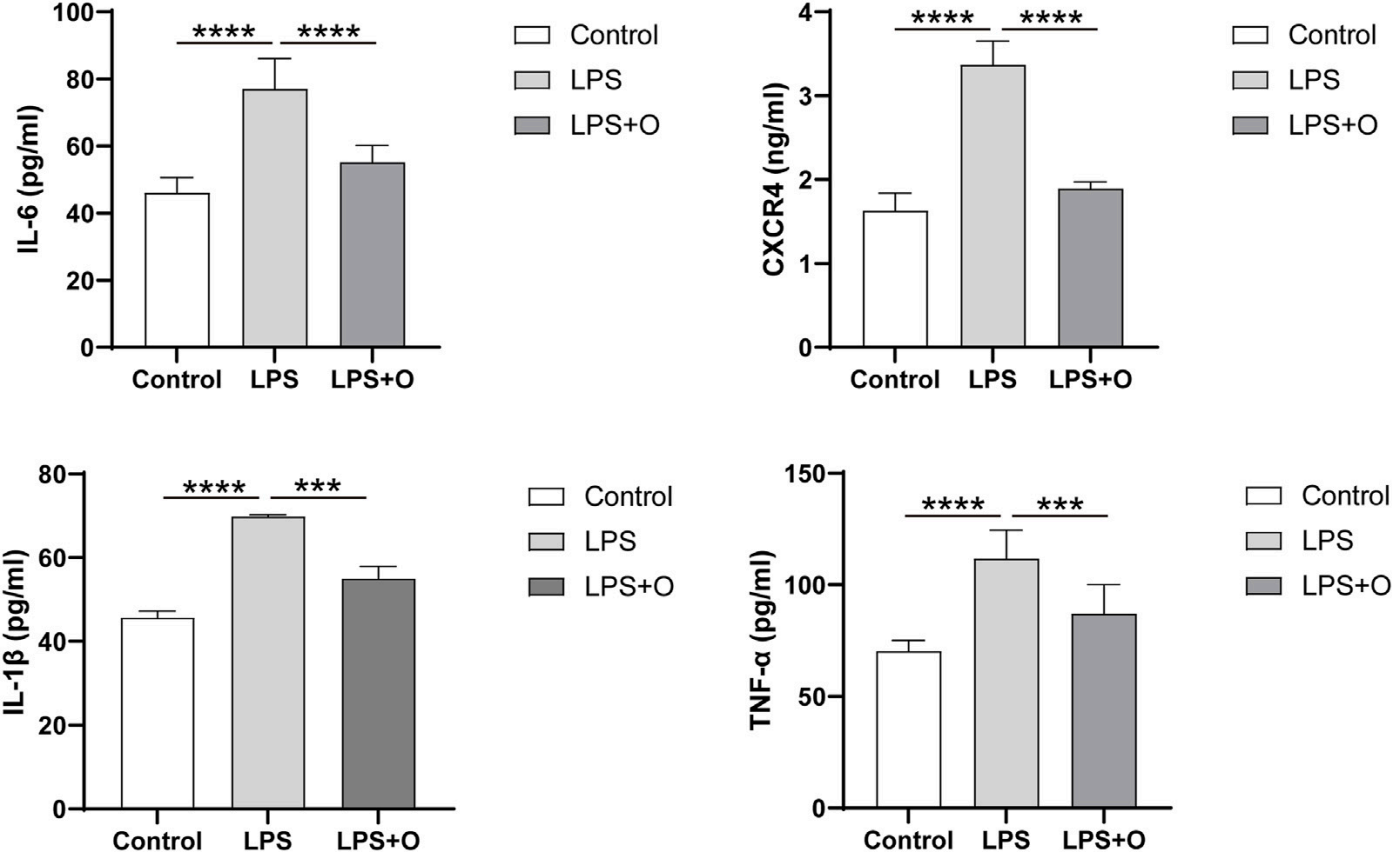
Ondansetron can decrease the inflammatory factors produced by LPS-stimulated neutrophils. Levels of inflammatory factors IL-6, IL-1β, TNF-α, and CXCR4 after being incubated at 37°C for 4 h in the cell supernatant of the control group, LPS group, and LPS + O group. Control, Neutrophils; LPS, Neutrophils stimulated by LPS; LPS + O, Neutrophils stimulated by LPS was treated with ondansetron. Results are presented as mean ± SD. Student’s t-test was used to compare means of two groups. (***P < 0.001, ****P < 0.0001).

Compared with the control group, LPS stimulation of neutrophils caused a significant inflammatory response of neutrophils with inflammatory factors IL-6, IL-1β, TNF-α, and CXCR4 being significantly increased. Treatment of neutrophils stimulated by LPS with ondansetron resulted in significantly decrease in IL-6, IL-1 β, TNF-α, and CXCR4 levels (Figure 7).

## Discussion

In this study, we found that the pathway of neutrophil extracellular traps (NETs) formation is statistically significantly involved in the action of ondansetron against AKI, sepsis, and ARDS. The inflammatory damage caused by the excessive formation of NETs likely has significant research value in the occurrence and development of critical illness. Ondansetron may affect *TLR8, NE, MPO, NFKB1, and MAPK14* to inhibit the formation of NETs, thereby reducing excessive inflammatory damage in the occurrence and development of critical diseases. Ondansetron and these primary predictive biotargets may potentially be used to treat critical illness in future clinical practice.

Ondansetron is a first generation 5-HT_3_ receptor antagonist and is widely used antiemetic drug in the clinic. A pharmacoepidemiology study reported that ondansetron was associated with a significant decrease in 90-day mortality on the High Density Intensive Care database containing intensive care data for 13 hospitals across Western (Gray et al., 2022). A respective study of 51,342 ICU patients also found that early ondansetron application is protective against in-hospital mortality in ICU patients(Fang et al., 2022). For critical patients with AKI and sepsis respectively, it is showed potentially favorable effects of ondansetron on reduced in-hospital mortality (Gray et al., 2022; Guo et al., 2021; Tao et al., 2021). For intensive care unit-admitted COVID-19 patients, administration of ≥8 mg of ondansetron within 48 h of admission was correlated with an adjusted hazard ratio for 30-day all-cause mortality (Bayat et al., 2021). In addition, ondansetron and other 5HTR3 antagonists was reported to have organ-protective effect on lung (Wang et al., 2019), liver (Gong et al., 2019; Liu et al., 2011), heart (Liu et al., 2019) and nervous system (Setoguchi et al., 2011) in rat sepsis or shock models.

Nets formation play a very important role in the development and progression of different critical diseases. For AKI, Raup-Konsavage et al. (2018) showed that the core substances of NETs—PAD4—plays a key role in the inflammatory response and tissue damage of ischemic AKI. Studies by Nakazawa et al. (2017) have indicated that renal tubular necrosis and the formation of NETs accelerate renal injury and distal organ dysfunction through the release of cytokines and histones. Targeting PAD4 and/or NETs may provide new therapeutic strategies for AKI (Liu and Dong, 2018). For sepsis, excessive activation of neutrophils and release of NETs may induce endothelial cells to transition to pro-inflammatory and pro-coagulant phenotypes (Zhang et al., 2023). Neutrophils and NETs can degrade the glycocalyx on the surface of endothelial cells and increase endothelial permeability, thus leading to impaired microcirculation blood flow, inadequate tissue perfusion, and life-threatening organ failure in the late stage of sepsis (Zhang et al., 2023). For ARDS, the formation of NETs was highly correlated with pulmonary vascular immunothrombosis, airway mucus secretion, and cytokines storm (Skendros et al., 2020). NETs released by neutrophils can promote lung epithelial cell death (Barnes et al., 2020).

Our study used network pharmacology to further understand the mechanism of ondansetron in the treatment of critical illness and found that ondansetron likely inhibits the formation of NETs, thereby reducing excessive inflammatory damage in the occurrence and development of critical diseases.

However, the specific underlying mechanism of ondansetron in the formation of NETs is yet unclear. Studies have shown that the application of 5-HT3 receptor antagonists is related to the decrease and inhibition of MPO, one of the core substances in the formation of NETs (Tsukamoto et al., 2017; Motavallian-Naeini et al., 2012), thus reducing the damage caused by inflammatory response. The effects of 5-HT3 receptor antagonists may be direct effects mediated by 5-HT3 receptors, but there may also be other pathways independent of 5-HT3 receptors, such as those acting on 5-HT1B receptors, 5-HT1C receptors, α7nACh receptors, p38 MAPK pathway, calcineurin, NF-κ B pathway, peroxisome proliferator activated receptor γ (PPAR- γ) (Zulkifli et al., 2019). Similar to the results of this study, many studies on the mechanism of ondansetron are focused on p38 MAPK and NF-κB signaling pathways. For example, in a mouse sepsis model, 5-HT3 receptor antagonists could effectively inhibit phosphorylated p38 accumulation and NF-κB transactivation in nuclear factor macrophages, reducing the inflammatory response in septic mice (Gong et al., 2019). Ondansetron alleviates liver injury through p38 MAPK-dependent pathway in a rat hemorrhagic shock model (Liu et al., 2011). Moreover, NETs information of neutrophils usually works by activating the NF-κB and p38 MAPK signaling pathway (Li G. et al., 2023; Wang et al., 2022; Zeng et al., 2024; Li J. et al., 2023).

The possible correlation between ondansetron, TLR8 and neutrophils has also been reported. It is investigated that the anti-inflammatory properties of mianserin, a serotonin (5HT) receptor antagonist, was able to inhibit the endosomal TLR8 in primary human cells and inhibited the spontaneous release of TNF and IL-6 from rheumatoid arthritis synovial membrane cultures (Sacre et al., 2008). Moreover, human neutrophils were activated by TLR8 agonists (Cassatella et al., 2020). In a study of cowhide moss, antimicrobial peptide LL37 was found to trigger TLR8/TLR13-mediated cytokine and NETs complex release *in vitro* and *in vivo*. Blocking TLR8 with inhibitory oligonucleotides can alleviate this triggering effect (Herster et al., 2020). Fungal nucleic acids have the potential to induce the release of NETs *in vitro*, and research has shown that TLR8 is involved in the process of inducing NETosis activation (Smolarz et al., 2021). Therefore, ondansetron is very likely to affect multiple targets of *TLR8, NFKB1, MAPK14, NE, and MPO* to inhibit the formation of NETs.

In future research, there are several points to pay attention to. Firstly, other commonly used clinical drugs were found also have the effect of inhibiting NETs, such as lidocaine (Ren et al., 2023; Zhang et al., 2022), alpha-linolenic acid (Liu et al., 2023), colchicine (Li et al., 2024) and enoxaparin (Córneo et al., 2023). It will be very meaningful to discuss the possibility of combining ondansetron with these drugs, and to explore whether this combination can further enhance the effect of inhibiting NETs formation and alleviating inflammatory responses, and the advantages of this combination therapy in the improvement of critical disease outcomes. Secondly, a 32 mg single intravenous dose of ondansetron may induce patients to develop QT interval prolongation and the fatal heart rhythm known as Torsades de Pointes (Sutherland et al., 2022). Drug manufacturers currently recommended that a daily dose of OND should not be more than 16 mg (Sutherland et al., 2022). Studies showed daily low-to-moderate dose (0–16 mg) of ondansetron application is protective against in-hospital mortality (Fang et al., 2022). However, for ICU patients with complicated conditions and multiple medications, further experiments *in vivo* and *in vitro* are necessary to confirm the optimal daily dose of OND. Thirdly, ondansetron’s protective effect against in-hospital mortality is more evident in ICU patients with cardiovascular diseases (Fang et al., 2022). NETs are considered new players involved in the development and progression of cardiovascular diseases, including coronary artery disease, its acute manifestations in particular acute myocardial infarction and peripheral artery disease along with ischemic stroke, atrial fibrillation and heart failure (Natorska et al., 2023). The role of ondansetron might vary in different patient groups, and guidance for personalized treatment of ondansetron deserves to be explored.

### Study strengths

AKI, sepsis, and ARDS, the three most common illnesses seen in the ICU are not specific diseases, rather can be caused by many pathogenic factors. These three syndromes often occur together or sequentially under certain etiological stimuli, and it is unclear whether they have a common pathogenesis. Through network pharmacology analysis of the intersection targets of ondansetron against the three syndromes, this study identified not only the disease–drug intersection targets but also the common pathogenesis of the three syndromes. Network pharmacology is a good method for studying whether “old clinical drugs” have new indications. Thus far to our knowledge, few study has yet targeted the effect of ondansetron on neutrophils and neither have there been any studies on ondansetron and NETs.

### Limitations

This study conducted a preliminary exploration on the effect of ondansetron on neutrophils in the inflammatory response at the cellular level. However, the current experimental results didn’t show the specific mechanism how it is reducing the inflammatory signaling. To confirm that ondansetron may improve organ function and outcomes by reducing the excessive inflamatory response of neutrophils and related mechanism, further animal experiments and clinical randomized controlled trials are needed. Our study based on the above scientific assumptions was funded by the Provincial Natural Science Foundation of China (NO. 2023A1515011988), and our team will further investigate the potential pharmacological effects of ondansetron on critical illness. Moreover, if an ondansetron-only group was added, the neutrophils experimental design would be more complete in this study.

## Conclusion

Our bioinformatics approach of using network pharmacology has effectively highlighted potential therapeutic targets and pharmacological pathways of ondansetron’s action against critical illness. The bioinformatics data presented identifies potential targets that should be validated in a preclinical study before being used for the clinical treatment of critical illness with ondansetron.

## Data Availability

The original contributions presented in the study are included in the article/Supplementary Material, further inquiries can be directed to the corresponding author.

## Abbreviations

AKI, Acute kidney injury; ARDS, Acute respiratory distress syndrome; ELISA, Enzyme linked immunosorbent assay; KEGG, Kyoto Encyclopedia of Genes and Genomes; NE, Neutrophil elastase; NET, Neutrophil extracellular traps; TCMSP, Traditional Chinese Medicine Systems Pharmacology.
