# Supplementary figures and images for: The therapeutic targets and signaling mechanisms of ondansetron in the treatment of critical illness in the ICU
Source: Front Pharmacol. 2024 Aug 21;15:1443169. doi: 10.3389/fphar.2024.1443169 (PMC11372243; doi:10.3389/fphar.2024.1443169)

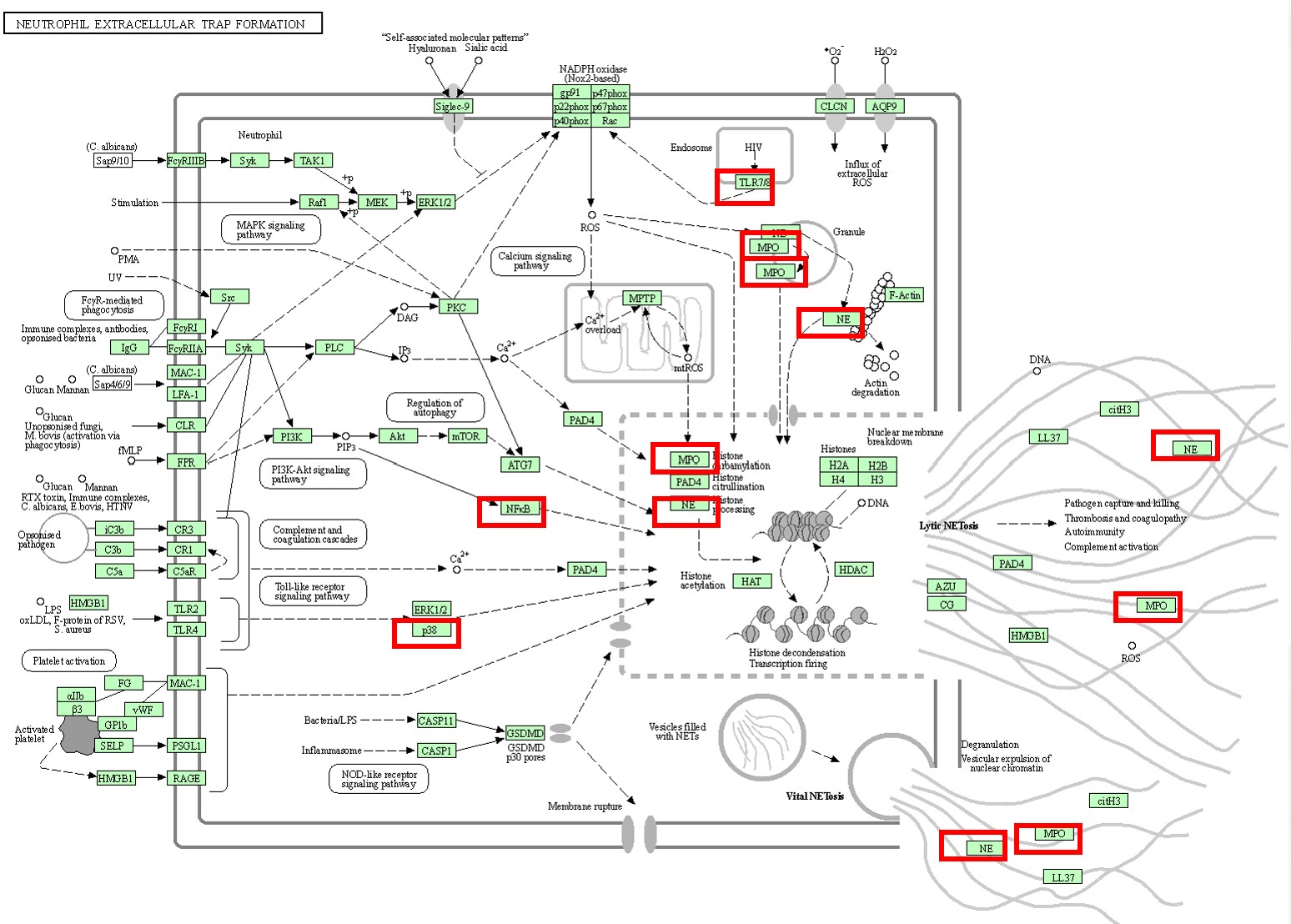

Supplement: Supplementary file 1 [file Image1.JPEG]

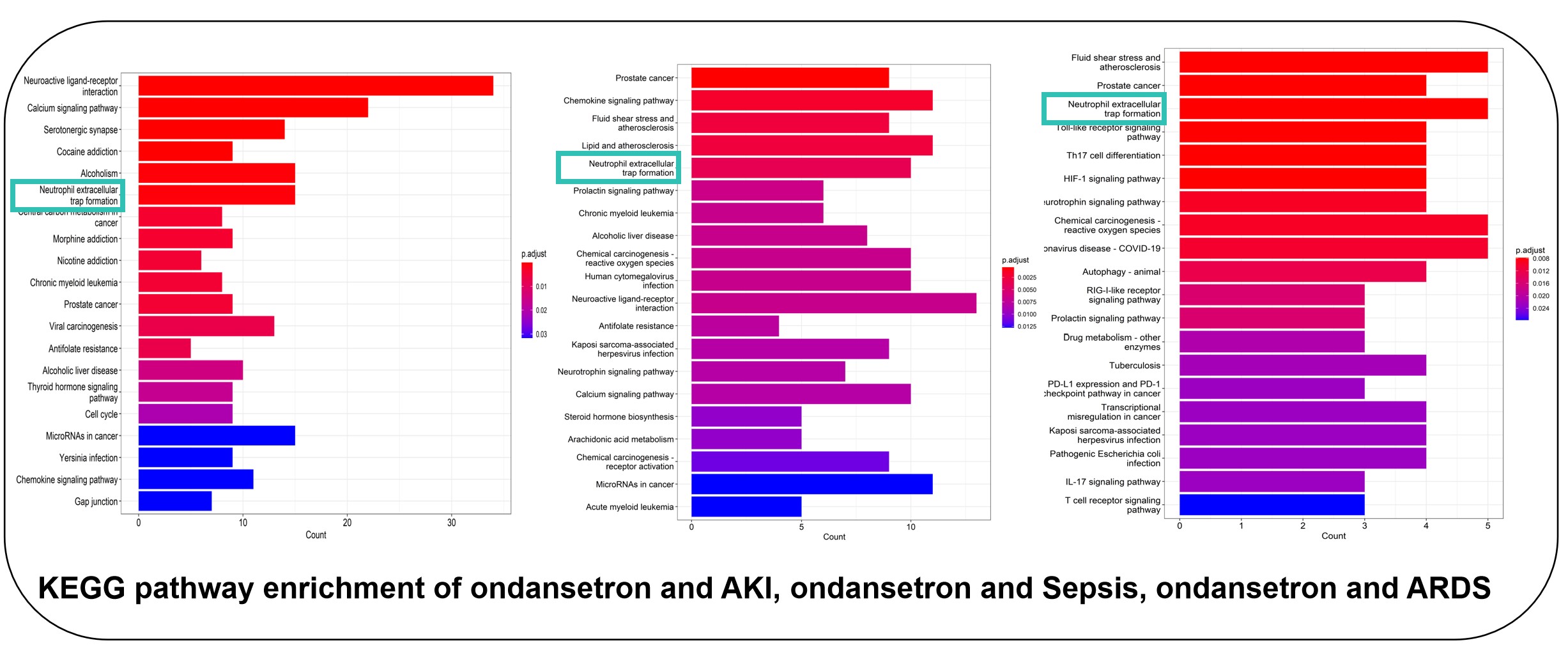

Supplement: Supplementary file 2 [file Image2.JPEG]
